# Cost-effectiveness of comprehensive geriatric assessment: systematic review of economic evaluations

**DOI:** 10.1093/ageing/afag203

**Published:** 2026-07-12

**Authors:** Amanuel Lulu Yigezu, Christina Hayes, Rose Galvin, Dominic Trépel

**Affiliations:** School of Medicine, Trinity College Dublin, Dublin, Leinster, Ireland; School of Medicine, School of Allied Health, Faculty of Education and Health Sciences, Ageing Research Centre, Health Research Institute, University of Limerick, Limerick, Ireland; School of Allied Health, Ageing Research Centre, Health Research Institute, University of Limerick, Limerick, Ireland; School of Medicine, Trinity College Dublin, Dublin, Leinster, Ireland; Trinity College Institute for Neuroscience, Trinity College Dublin, Dublin, Ireland

**Keywords:** comprehensive geriatric assessment, economic evaluation, systematic review, older people

## Abstract

**Background:**

Comprehensive geriatric assessment (CGA) can be resource-intensive as its delivery involves multidisciplinary personnel, multidimensional assessment, care planning and follow-up. The aim of this systematic review is to appraise and synthesise evidence on the cost-effectiveness of CGA across settings.

**Methods:**

Full economic evaluation studies examining CGA were searched across five databases. We included studies involving adults aged ≥65 years who were identified as living with frailty or at risk of adverse outcomes. Two independent reviewers screened studies against eligibility criteria and extracted data using a pretested extraction form. Reporting quality was assessed using the Consolidated Health Economic Evaluation Reporting Standards framework. We reported the results through narrative summaries, tables and figures.

**Results:**

A total of 4864 studies were identified, of which 17 were included across 7 settings (11 community, 6 hospital). Of 13 cost-utility analysis studies, incremental quality-adjusted life years ranged from −0.06 to 0.54 and incremental costs ranged from €−12 744 to €29 785. The probability CGA was cost-effective ranged from 0% to 98.9% across stated willingness-to-pay thresholds (cost-effective in 5/13, not cost-effective in 7/13, 50% chance in 1/13). Two studies showed that cost-effectiveness can vary by frailty severity and residential status. The resource intensity of CGA varied, ranging from 25% (case management protocol) to 81% (comprehensive assessment). Only five studies accounted for informal caregiver costs. The reporting quality ranged from 38% to 90%.

**Conclusion:**

The cost-effectiveness of CGA remains uncertain. Variations in resource intensity of CGA, frailty severity, residential status of older adults and setting may explain this heterogeneity.

**PROSPERO registration no:**

CRD42023492586.

## Key Points

- Considerable variability exists in comprehensive geriatric assessment (CGA) resource intensity; using the Template for Intervention Description and Replication checklist can improve its standardisation.
- The cost-effectiveness of CGA is uncertain. Cost-effectiveness of CGA may differ by frailty severity, baseline residential status and baseline health risks. Excluding informal care costs may result in a failure to capture the most likely economic impact of CGA. Adherence to the CHEERS will serve to improve the transparency and reliability of economic evaluations.

## Background

Comprehensive geriatric assessment (CGA) is a multidimensional, interdisciplinary diagnostic and therapeutic process focused on determining an older person’s medical, psychological and functional capability to develop a coordinated care plan [1]. The key defining characteristics of CGA include its delivery by a team of healthcare professionals with specialist geriatric expertise, a comprehensive assessment extending beyond medical treatment, multidisciplinary team (MDT)-based decision-making, follow-up and active involvement of older adults in decision-making [2].

Several systematic reviews have evaluated the clinical effectiveness of CGA across settings. In hospital environments, existing reviews encompass acute care hospitals (including emergency departments and acute geriatric units) [3–5] and inpatient care [2, 6], as well as specific populations such as surgical patients [7–9], cancer patients [10, 11] and the prevention of delirium [12]. Meanwhile, a growing number of reviews have focused on community-based contexts, including primary care [13, 14], community settings [1, 15] and long-term care facilities [16].

As CGA can be resource-intensive, health systems are not only interested in understanding the clinical effectiveness of CGA but also in assessing its value in terms of delivery costs and its downstream impact on health service resource utilisation and the health gains [often expressed as Quality Adjusted Life Years (QALYs)]. This economic framework ensures resource allocation decisions are efficient and do not displace resources from other interventions that may yield better health benefits to the population. Economic evaluations support decision-makers in understanding the value (health benefit and resource use consequences) of spending on CGA when compared to other health interventions, such as standard care [17].

Recent systematic reviews have incorporated economic aspects of CGA within broader clinical effectiveness studies. For example, a systematic review by Briggs *et al.* [1] described the costs of CGA in community settings, and Garrard *et al.* [13] reported cost-effectiveness outcomes as secondary analyses. However, these reviews did not evaluate full economic evaluations or appraise how costs and outcomes were identified, measured and valued. Given that decisions about implementing CGA should depend on robust economic evaluations, a targeted review is necessary to assess and critically evaluate the existing evidence base. Therefore, the aim of this systematic review is to appraise and synthesise published evidence on the full economic evaluation studies of CGA.

## Methods

This review follows the 2020 PRISMA guidelines [18]. The review is registered in Prospero with registration no CRD42023492586, and the protocol is published in HRB Open Research [19].

### Economic evaluation framework

Economic evaluation can be classified according to scope, and design. In terms of its scope, economic evaluation can be classified into partial and full economic evaluation [20]. Partial economic evaluation evaluates only one dimension, either the cost or the effectiveness of alternative interventions or examines both the cost and effectiveness of a single intervention. Full economic evaluations compare both the costs and the effectiveness of two or more alternatives [20, 21]. The total cost of each alternative is the sum of the costs of delivering the interventions with resource-use consequences (e.g. health service utilisation) that occur after the intervention is delivered.


$$ Tc= Ci+ Cc $$

where: *Tc*: Total cost; *Ci*: cost of intervention; *Cc*: resource-use consequence

Full economic evaluations can be further classified according to how effectiveness is measured and valued. Cost-utility analysis (CUA) measures the effectiveness in QALYs; cost-effectiveness analysis (CEA) reports the effectiveness in natural units; cost-consequences analysis reports multiple effectiveness outcomes; cost–benefit analysis expresses the effectiveness in monetary terms; and cost-minimisation analysis assumes the interventions are equally effective and only compares the costs [20, 21].

Economic evaluations are most commonly conducted from a healthcare, health and social care or societal perspective. The healthcare perspective includes costs incurred by the healthcare system. The health and social care perspective includes additional social service costs to the healthcare perspective. The societal perspective encompasses both the costs incurred by patients and their caregivers and health and social care systems [20]. With respect to design, economic evaluations can be classified as trial-based or model-based. Trial-based economic evaluation is conducted alongside a randomised controlled trial by tracking relevant costs and effectiveness data of comparison groups throughout the trial period. Model-based economic evaluations define a set of multiple factors within a single decision-analytic framework, generating expected costs and effects for comparison groups [20].

### Search strategy and selection criteria

To identify relevant studies, a search strategy was developed with support from a librarian at Trinity College Dublin. This search strategy was applied across various databases, including Embase, MEDLINE, CINAHL, NHS EED and Tufts CEA Registry, in accordance with the recommendations of the Centre for Reviews and Dissemination Guidance [22]. The search strategy is provided in Appendix 1, Text 1. EndNote V21 was used to import studies. Covidence was used to manage search results and remove duplicate records. Database searches were conducted on 13 September 2025.

Study selection was outlined using a PRISMA flow diagram. Two reviewers (A.Y. and C.H.) independently screened eligible titles/abstracts. The full texts of eligible studies were reviewed, and the decision to include them was made independently by two authors. Disagreements regarding the inclusion of studies were resolved by a third or fourth author (D.T. or R.G.).

### Criteria for eligibility of studies

Table 1 presents the inclusion and exclusion criteria.

**Table 1:** Inclusion and exclusion criteria.

Criteria	Inclusion	Exclude
Population	Older adults 65 years or above. Identified as either living with frailty or at risk of adverse outcomes	Populations with specific diseases (e.g. CGA for patients with cancer) were excluded to improve comparability across studies and enhance generalisability to routine frailty-focused geriatric care. Population not identified as either living with frailty or at risk of adverse outcomes.
Intervention	Comprehensive geriatric assessment	
Comparator	- Care as usual, standard care or do-nothing	Head-to-head comparison. Example CGA at inpatient versus CGA at home.
Outcome	Outcomes: average cost, average effectiveness, incremental cost and incremental effect	All outcomes from partial economic evaluations
Study types	Full economic evaluations (model-based or trial-based).	Partial economic evaluation (cost-analysis, cost-description, outcome description) Commentaries, Methodological articles
Setting	All settings (inpatient, outpatient, General Practice, Home-based, Emergency, long-term facilities and others).	

### Data extraction and management

Two reviewers (A.Y. and C.H.) independently extracted data from the selected studies using a structured data collection form on Covidence. Disagreements between the two reviewers were discussed with the third and fourth reviewers (D.T. or R.G.).

### Data synthesis and analysis

Study characteristics and results are presented in tables and figures. The resource intensity of CGA was described in terms of its components (activities) and the personnel involved. The components were categorised based on the systematic review by Ellis *et al.*, which identified the most commonly reported activities when CGA is implemented [2]. These include comprehensive assessments, MDT meetings, goal setting, use of assessment tools, protocols guiding the management of key conditions, home or ward environment, follow-ups, patient involvement in decision-making and MDT compositions. Resources used to estimate the costs are also presented. For CUA studies, we adjusted all costs to 2024 euros (€) and presented the cost-effectiveness results in a table [23].

### Appraisal of reporting quality

Two reviewers (A.Y. and C.H.) independently assessed the reporting quality, and the third and fourth authors (D.T. or R.G.) were involved in resolving disagreements between the two reviewers. The Consolidated Health Economic Evaluation Reporting Standards (CHEERS) framework [24] was used to assess reporting quality. We employed a traffic-light colour-coding system to visually represent the degree to which key elements of the CHEERS framework were addressed: green for fully addressed; yellow for partially addressed; red for not addressed; and black for not applicable. Items were scored as 1 (fully addressed), 0.5 (partially addressed) or 0 (not addressed). The percentage of reported CHEERS items was presented within and across studies.

## Results

**Figure 1:**
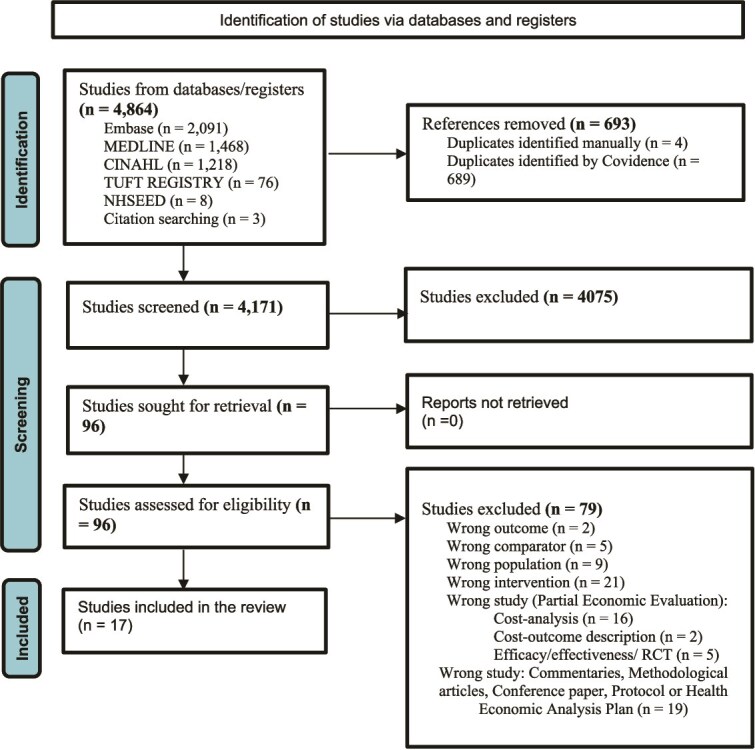
PRISMA flow diagram for the identification of studies.

The database searches identified 4864 studies. Removing 693 duplicates, title and abstract screening was conducted for 4171 studies. After excluding 4075 studies, 96 studies were included for a full-text review. Of the 96 studies, 17 were included in the review. Figure 1 presents the study flow diagram.

### General characteristics of the studies

Among the included studies, three were conducted in general practice [25–27], six in the home environment [28–33], two in emergency departments [34, 35], three in inpatient departments [2, 36, 37] and one each in an outpatient department [38], residential home [39], and a community-based clinic [40]. The studies were published between 1999 and 2022. Sixteen studies were conducted in high-income settings, five of which were in the Netherlands [26, 28, 31, 32, 39] (Appendix 1, Supplementary Fig. S1).

In terms of perspective, two studies reported both societal and healthcare perspectives [25, 27], six adopted a societal perspective [29, 31, 32, 35, 37, 39], two applied health and social care perspectives [33, 34] and five adopted a healthcare perspective [2, 26, 28, 30, 38]. Two studies did not specify the perspective [36, 40].

In 12 studies, the time horizon varied from 6 months to 5 years. The time horizon was less than 6 months in three studies, with a duration of 1 month in one study [37] and 3 months in two studies [35, 36] (Table 2).

**Table 2:** General characteristics of the studies.

Characteristics	Description of characteristics	Number of studies	References
Year of publication	1999–2005	1	[40]
	2006–2011	3	[28, 36, 37]
	2012–2017	6	[2, 29–32, 34, 39]
	2018–2025	7	[25–27, 33, 35, 38]
Country of study	Australia	2	[27, 30]
	Indonesia	1	[36]
	Finland	1	[37]
	Germany	1	[29]
	Netherlands	5	[26, 28, 31, 32, 39]
	Scotland	1	[33]
	Sweden	3	[25, 35, 38]
	UK	2	[2, 34]
	USA	1	[40]
Target population by age	Older than 60	1	[36]
	Older than 65	5	[2, 32, 33, 37, 40]
	Older than 70	6	[26–28, 30, 31, 34]
	Older than 75	3	[25, 35, 38]
	Older than 80	2	[29, 39]
Care settings	Home-based	6	[28–33]
	Inpatient	3	[2, 36, 37]
	Outpatient	1	[38]
	General practice	3	[25–27]
	Emergency	2	[34, 35]
	Community-based clinic	1	[40]
	Residential home	1	[39]
Study design	Trial-based	14	
	Model-based	3	[2, 36, 38]
Perspective of the study	Societal and healthcare	2	[25, 27]
	Societal	6	[29, 31, 32, 35, 37, 39]
	health and social care	2	[33, 34]
	Healthcare	5	[2, 26, 28, 30, 38]
	Perspective not reported	2	[36, 40]
Time horizon	1 month	1	[36]
	3 months	2	[34, 35]
	6 to 9 months	4	[27, 28, 33, 39]
	12 to 18 months	4	[26, 29, 30, 37]
	2 to 5 years	4	[25, 31, 32, 40]
	>5 years	1	[38]
	Not reported	1	[2]
Subgroup analyses	Yes	2	[30, 34]
	No	15	
Analyses of uncertainty	Deterministic sensitivity analysis	2	[2, 26]
	Bootstrapping	13	[25, 27–35, 37, 39, 40]
	Probabilistic sensitivity analysis	1	[38]
	Not reported	1	[36]
Source of funding	Public	12	[2, 25–28, 30, 32–35, 38, 39]
	Not reported	5	[29, 31, 36, 37, 40]

### Resource intensity for the configuration of models of CGA

Fifteen studies reported conducting a comprehensive assessment and a MDT meeting, and in 13 studies, older adults received a follow-up visit after the initial assessment. Eight studies implemented goal setting and patient consultation in decision-making. Improving the home or ward environment was reported in 11 studies. The use of assessment tools and protocols was documented in four studies. The most frequently reported MDT members were nurses (reported in 16 studies), followed by physiotherapists [9], and then consultant geriatricians, occupational therapists and social workers (6 each). Across studies, the median number reporting each CGA characteristic was eight (range: 4–13), whereas the median number reporting each personnel category was four (range: 1–14). Supplementary Figure S2  in  the  Appendix  1 presents the resource intensity of CGA across settings.

### Key resources measured in cost estimation

To estimate the costs of the comparison groups, 14 studies (82%) included the costs of delivering CGA. From the downstream healthcare resource utilisation, inpatient visits were reported in 16 studies (94%), outpatient care in 11 (65%) and general practice use in seven (41%). Among social care costs, nursing home-related resource use was reported in eight studies, followed by home visits [6], temporary residences [3] and residential homes [2]. From studies that adopted a societal perspective, informal caregiver costs were considered in five studies. Two other studies considered the costs of home help services and nursing home care as societal costs [25, 35], and one study did not explicitly define what constitutes a societal cost [37]. Key resources measured across settings are presented in Appendix 1, Supplementary Fig. S3.

### Measurement and valuation of outcomes

Out of the 17 studies, effectiveness outcomes were measured in QALY only in 5 studies (CUA), only non-QALY outcomes in 3 studies (CEA), and both QALY and non-QALY outcomes in 9 studies.

QALYs were estimated using EQ-5D in nine studies. Four other studies used the Health Utility Index-3 [35], the Short-Form Six-Dimension (SF-6D) [39], the Quality of Well-Being Scale [40] and EQ-5D + 3 L [26]. One study estimated QALYs by transforming evidence from the Barthel index [2].

Effectiveness measures beyond QALYs included transition from frailty [30], successful treatment [28], measures of functional (in)dependence [26, 27, 31, 32, 37, 39, 40], quality-adjusted life days [36], quality indicator scores [39], health-related quality of life (HRQoL) [32] and life years living at home or life years gained [2]. A description of the outcomes is presented in the Appendix 1, Supplementary Table S1.

### Cost-utility analysis of CGA

Thirteen studies reported economic evaluation outcomes using QALYs (CUA). Overall, seven studies concluded that CGA is not cost-effective, five found it to be cost-effective, and one reported a 50% probability of cost-effectiveness at stated willingness-to-pay threshold.

In community settings, nine CUAs were identified. Home-based CGA was evaluated in five studies: four found no change in average incremental QALY and suggested CGA was not cost-effective [29–32], whereas one study with incremental costs of −€4474.20 (95% CI: −€8549.5 to €393) and QALY difference of −0.002 (95% CI: −0.013 to 0.001) was considered cost-effective [33]. For general practice-based CGA, two studies showed conflicting results. The first study showed that CGA was cost-saving, with an incremental cost of €−12 744.42 (95% CI: €−25 157.07—€−462.42) and an incremental QALY of 0.05 (95% CI: −0.17 to 0.08) [25]. Conversely, the second study reported increased costs of €2179.82 (95% CI: €−6399.02 to €2039.37) and an incremental QALY of −0.03 (95% CI: −0.10 to 0.00), and suggested that CGA was not cost-effective [26].

In a hospital setting, four CUA studies were identified. In emergency department (ED) settings, one study reported that CGA dominated usual care, resulting in cost savings of €−3333.42 and an incremental QALY of 0.0242 [35]. In contrast, another ED-based study found CGA not cost-effective with incremental costs of €484.0 (95% CI: €309.3 to €657.1) and incremental QALY of −0.001 (95% CI: −0.009 to 0.007) [34].

In Table 3, the cost-effectiveness of CGA is presented according to several key features, including care setting, resource intensity (components and personnel involved), costing perspective (including informal caregiver costs) and time horizon. The cost-effectiveness plane is illustrated in Appendix 1, Supplementary Fig. S4.

**Table 3:** Cost-effectiveness of CGA compared to usual care

Author, country	CGA component (n/8), personnel types involved	Setting	Incremental QALY mean (95% CI)	Perspective	Total incremental cost in €^p^ Mean (95% CI)	ICER (€^p^QALY)	Reported WTP per QALY	Probability of being cost-effective at WTP
VanLeeuwen 2015, Netherlands	7/8, 2	Home-based	0.003 (−0.006; 0.012)	Societal^**+**^	490.2 (−672.0; 1561.5)	163 400	US$0 to 30 000	20%–25%
				Healthcare	312.5 (−669.21; 319.1)	104 166.6		26%–33%
Brettschneider 2015, Germany	4/8, 5	Home-based	0.0061 (0.0388)^*^	Societal^**+**^	6612.2 (4537.2)^*^	NR	€ 50 000	15%
Fairhall 2015, Australia	5/8, 7^**G**^	Home-based	−0.022 (−0.088; 0.46)	Healthcare	1563.3 (−4152.7; 7449.2)	NR	NR	10.8%^C^
Metzelthin 2015, Netherlands	6/8, 4	Home-based	−0.06 (−0.14; 0.02)	Societal^+^	8213.3 (873.35; 17 298.6)	NR	NR	2%
Singh 2022, Scotland	4/8, 7^**G**^	Home-based	−0.002 (−0.013, 0.010)	Societal^+^	−4474.20 (−8549.5; 393)	NR	£20 000	98%
				Health and social care	−3777.2 (−7502.5; 50.4)			97%
Keeler 1999, USA	7/8, 5^**G**^	Community-based clinic	0.07	NR	1094.1 (762.2)^*^	15 630.0	NR	NR^**^
Nord 2022, Sweden	3/8, 2	General practice	0.05 (−0.17; 0.08)	Societal	−12 744.4 (−25 157.07;462.4)	Dominant^C^	NR	77.9%^C^
				Healthcare	−6806.8 (−1289.6;12 325.2)	Dominant^C^		78.1%^C^
Ruikes 2018, Netherlands	6/8, 5	General practice	−0.03 (−0.10; 0.00)	Healthcare	−2179.8 (−6399.0; 2039.4)	NR	€ 0 to €100 000	NR
MacNeil Vroomen 2012, Netherlands	6/8, 3	Residential home	0 (−0.01; 0.01)	Societal^**+**^	578.3 (304.1; 1179.5)	−354 571.1	€ 0 to €100 000	14%
Lundqvist 2018, Sweden	6/8, 6	Outpatient	0.54	Healthcare	29 785.4	55 158.1	€ 50 000	60%
Ekerstad 2018, Sweden	5/8, 4	Emergency	0.0242	Societal^**T**^	−3333.4	Dominant^C^	NR	98.6%^C^
				Healthcare	−2427.2	Dominant^C^	NR	98.9%^C^
Tanajewski 2015, UK	1/8, 5^**G**^	Emergency	−0.001 (−0.009; 0.007)	Societal^**T**^	484.0 (309.3; 657.1)	Dominated^E^	£20 000	0%
Ellis 2017, UK	NA	Inpatient	0.012 (−0.024; 0.048)	Healthcare	367.0 (−225.9; 949.0)	30 583.33	£20 000	50%

^**G**^geriatrician involved ^*^Standard error; ^**^Comparable to other approved interventions; ^**C**^less costly and more effective; ^**E**^more costly and less effective; ^**p**^2024 price year; ^**+**^Informal care cost considered, ^**T**^time horizon <6 months, NA, not applicable (model-based); NR, not reported; WTP, willingness to pay.

Subgroup analyses were conducted in two studies [30, 34]. Subgroup analysis from the first CEA study that used transition from frailty as an outcome showed that the intervention was deemed both cost-saving and more effective (transitioned more people out of frailty) for groups living with severe frailty [30] than for groups with frailty. The second study, conducted in an emergency care setting, evaluated CUA of CGA and found that the intervention was cost-effective for adults living in care homes but not for the non-institutionalised population [34].

### Reporting quality of included studies

**Figure 2:**
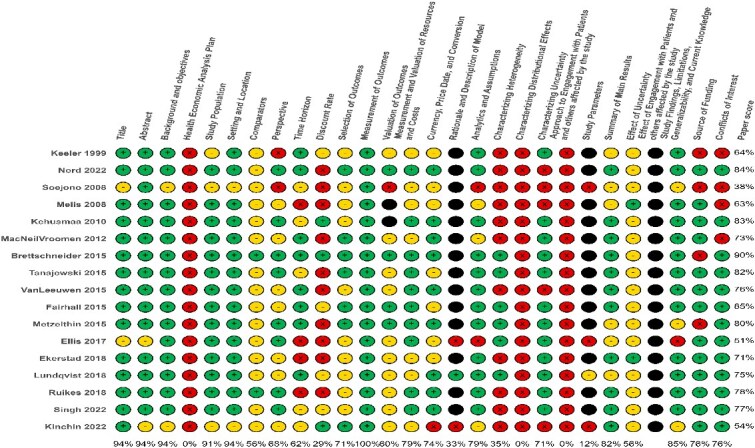
Traffic light system of the reporting quality of the studies using the CHEERS framework.

The reporting quality ranged from 38% to 90%, with 52.9% of studies meeting 76% or more of CHEERS reporting standards, 41.2% meeting 51 to 75% of the standards and 5.9% meeting less than 50% of all requirements. The least frequently reported items were study parameter (12%) and discount rate (29%). Seven CHEERS items were reported by less than 50% of the studies. Only six items were reported in more than 90% of the studies. Figure 2 presents reporting quality using a traffic-light system.

## Discussion

This systematic review identified 17 full economic evaluation studies, descriptively appraised and synthesised existing evidence on the cost-effectiveness of CGA. The cost-effectiveness of CGA remains uncertain and varies considerably across studies. This heterogeneity could be due to variation in resource intensity of CGA, including differences in components and personnel involved, frailty severity and residential status of older adults, and the setting where CGA is delivered.

Substantial variation exists in resource intensity of CGA. Comprehensive assessment and MDT meetings (81% each) were the most commonly used components, while protocols (25%), assessment tools (31%), goal setting (38%) and patient consultation (44%) were the least commonly implemented. Among the MDTs delivering CGA, nurses were involved in 88% of the teams, followed by physiotherapists (56%). In 38% of the included studies, consultant geriatricians, social workers and occupational therapists were involved. No study reported all CGA elements. This heterogeneity reflects the lack of standardised operational guidance for CGA or incomplete reporting of intervention details, limiting our ability to assess its value for money. Although the personnel involved may vary across care settings, standardisation of the resources and processes required to deliver CGA would improve comparability across studies and further strengthen the evidence base. Consistent use of the Template for Intervention Description and Replication (TIDieR) checklist would enhance transparency, replicability of interventions and reduce research waste [41], while supporting the decomposition of intervention components for more accurate cost estimation and evaluation of value for money [42].

The perspective chosen for economic evaluations should capture the resources associated with the costs driving the cost-effectiveness results. Of the eight studies conducted from a societal perspective (47%), five (29%) included informal caregiver costs [29, 31–33, 39]. The remaining three studies omitted the costs [25, 35, 37], with two defining nursing home care and home help as societal costs [25, 35]. Yet existing evidence shows that informal care costs are higher for older adults admitted to nursing homes [43]. A recent observational study in Sweden estimated the societal annual cost of informal care at €1150 million for community-dwelling older adults. These costs were €7477 per older adult living with frailty [44]. Therefore, omitting informal caregiver costs could lead to underestimating the actual societal costs of frailty. Clinical evidence suggests that CGA can improve functional status, optimise medication management, enhance care coordination and help delay institutionalisation [1, 2, 6, 14]. Although these improvements may reduce the burden on informal caregivers, the evidence from the five studies shows that the impact is uncertain. To evaluate how these changes in clinical outcomes affect the time and support required from informal caregivers, caregiver costs should be appropriately measured and valued. Detailed methods for measuring and valuing informal caregiver burden costs are presented in Appendix 1, Supplementary Table S2.

The instrument used to estimate QALYs should reflect outcomes that matter to older adults. In this review, QALYs were estimated using the EQ-5D HRQoL measure in nine studies. For interventions targeting older adults, the estimation of QALYs using the HRQoL instruments has been debated. Some studies suggest adopting a broader well-being measure to capture outcomes beyond existing HRQoL [45]. Supporting this view, a recent study compared ICECAP-O (ICEpop CAPability measure for Older people) and EQ-5D in a CEA of integrated care for frail older adults. The authors concluded that ICECAP-O showed a higher probability of cost-effectiveness than EQ-5D, with a lower risk of falsely claiming cost-effectiveness [46]. The recently revised Health Technology Assessment guideline of the Netherlands also calls for a new generic, preference-based outcome measure tailored to well-being to evaluate the cost-effectiveness of interventions [47]. As CGA is a diagnostic and therapeutic process aimed at improving or maintaining multidimensional aspects of older adults, it serves a purpose beyond simple medical management [48]. More studies are required to evaluate the sensitivity of the existing HRQoL measure in fully capturing these outcomes.

Time horizon is particularly important when evaluating CGA, as its effects on health outcomes and costs may emerge over extended periods. In economic evaluation, the time horizon should be long enough to capture the full impact of interventions, i.e. the most important difference between the costs and consequences [49]. In this review, time horizon ranged from 6 months to 5 years in 12 studies and exceeded 5 years in one study. Three studies had a time horizon of less than 6 months: two with a 3-month time horizon [34, 35] and one with a 1-month time horizon [36]. For CGA, a 3-month horizon is very short. A recent review recommends at least 6 months of follow-up to capture the impact of CGA interventions [1]. A similar review suggested that the impact of CGA on decline in mortality was greater at 3-year follow-up [50]. A study by Van Leeuwen *et al.* also reported that, after delivering CGA, societal costs were lower between months 18 and 24 than in the initial period [32].

The current review indicates that the number of studies in community settings (nine CUA studies; median = 1.5 per setting) is higher than in hospital settings (four studies; median = 1 per setting). This may reflect shifts in the health systems from hospital to community-centred models of care [1]. In addition, the probability that CGA is cost-effective varied across and within settings.

The CHEERS framework assessed the reporting quality of the studies according to the CHEERS-2022 reporting guideline. In this review, no study fully complied with the reporting standard. In all included studies, the health economic analysis plan, the evaluation of distributional effects, the approach to engagement with patients and others affected by the study, and the effect of such engagement were not reported. This is due to additional items included in the updated CHEERS statement in 2022 [49].

Characterising heterogeneity is one of the 28 items of the CHEERS guideline. Of the six studies that reported on characterising heterogeneity, population subgroup analyses were conducted in two [30, 34]. The findings suggest that the cost-effectiveness of CGA can vary by population characteristics, such as baseline institutional status and frailty severity. Therefore, adhering to the CHEERS reporting guideline not only improves the transparency of studies, but also enhances the interpretability and usefulness of the evidence base for decision-making.

Our study has several strengths. We critically examined the processes of identifying, measuring, and valuing costs and effects, thereby assessing the reliability of existing economic evaluations and providing guidance on key methodological issues for future research. Because unit costs vary across countries, describing the resource intensity of CGA provides a more meaningful basis for comparison. Additionally, we used the CHEERS framework to evaluate the reporting quality of the studies, supporting an assessment of the completeness of economic evaluation reports. However, this study also has limitations. Because countries use different cost-effectiveness thresholds, the probability that CGA is cost-effective may vary across studies. As there is no universally accepted or validated tool for assessing methodological quality, we did not formally assess the methodological quality of the included studies. Instead, methodological features were appraised descriptively.

In conclusion, this systematic review indicates that the cost-effectiveness of CGA remains uncertain. This uncertainty may result from heterogeneity in the resource intensity of CGA, including variation in number of components and personnel involved. This may limit the extent to which the findings can be interpreted as reflecting a single, standardised intervention. Cost-effectiveness also varied within and across settings. Furthermore, the inconsistent inclusion of informal caregiver costs may limit comparability across studies. Finally, differences in frailty severity, health risks and institutional status at baseline may also explain variation in the cost-effectiveness of CGA. Standardising CGA’s operational delivery and reporting it in accordance with the TIDieR checklist can strengthen the evidence base in geriatric care by clearly defining what is delivered. Future studies should adhere to the CHEERS reporting guideline.

## Supplementary Material

Supplementary_materials_afag203
