# Supplementary material for: Cost-effectiveness of comprehensive geriatric assessment: systematic review of economic evaluations
Source: Age Ageing. 2026 Jul 12;55(7):afag203. doi: 10.1093/ageing/afag203 (PMC13356896; doi:10.1093/ageing/afag203)
Supplement: Supplementary_materials_afag203 [file supplementary_materials_afag203.zip › Supplementary_materials_afag203.docx]

**Supplementary data**

**Cost-Effectiveness of Comprehensive Geriatric Assessment: Systematic Review of Economic Evaluations**

**Appendix 1. Supplementary data**

Contents

[Figure S1: Characteristics of studies by care setting, publication year and country. 3](#_Toc232275206)

[Figure S2: Description of characteristics of the CGA intervention by setting 4](#_Toc232275207)

[Figure S3: Measurements of health service utilisation for comparison groups by setting 5](#_Toc232275208)

[Figure S4: Cost-effectiveness plane for CGA intervention across settings compared to usual care, € 2024. 6](#_Toc232275209)

[Table S1: Characteristics of the studies 7](#_Toc232275210)

[Table S2: Valuation and measurement methods of informal care cost 10](#_Toc232275211)

[Supplementary Text 1: Search strategy 11](#_Toc232275212)

**
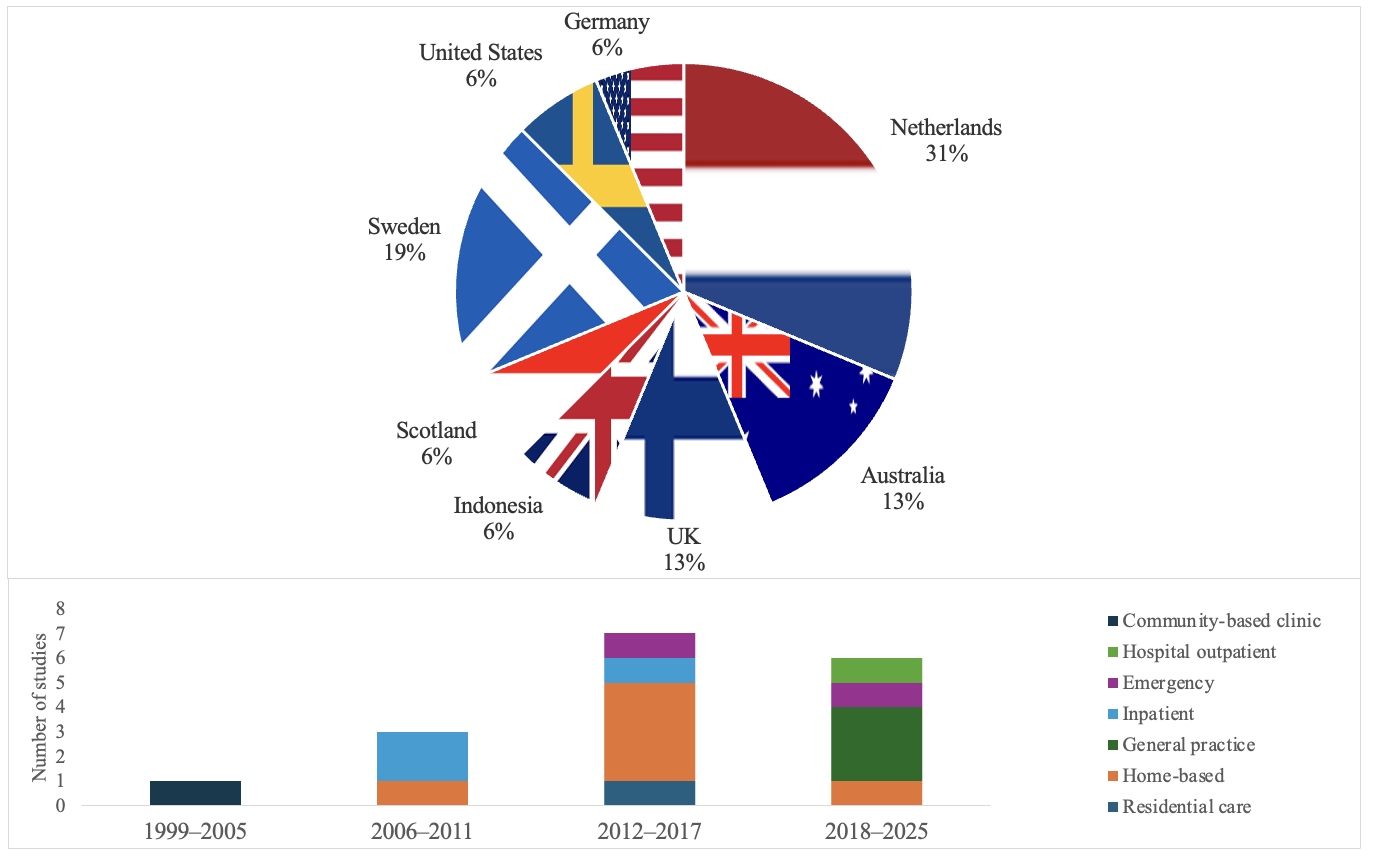
**

Figure S1: Characteristics of studies by care setting, publication year and country.

Alt text: Pie and stacked bar charts showing the distribution of included studies by country and publication period, with most studies conducted in the Netherlands and increasing publications after 2012 across multiple care settings.


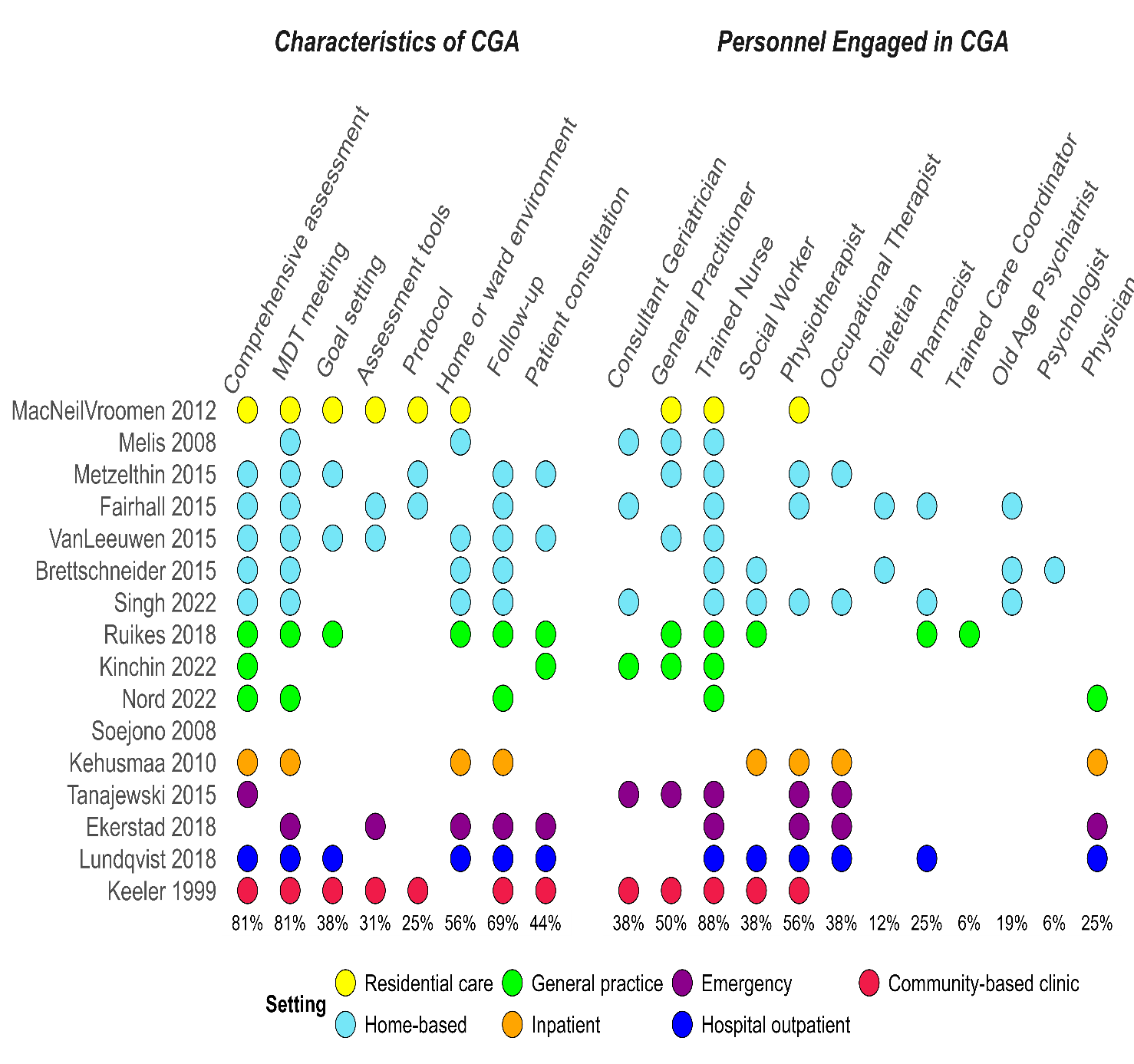


Figure S2: Description of characteristics of the CGA intervention by setting

Alt text: A graph showing variation in Comprehensive Geriatric Assessment components and multidisciplinary personnel across included studies, grouped by care setting.


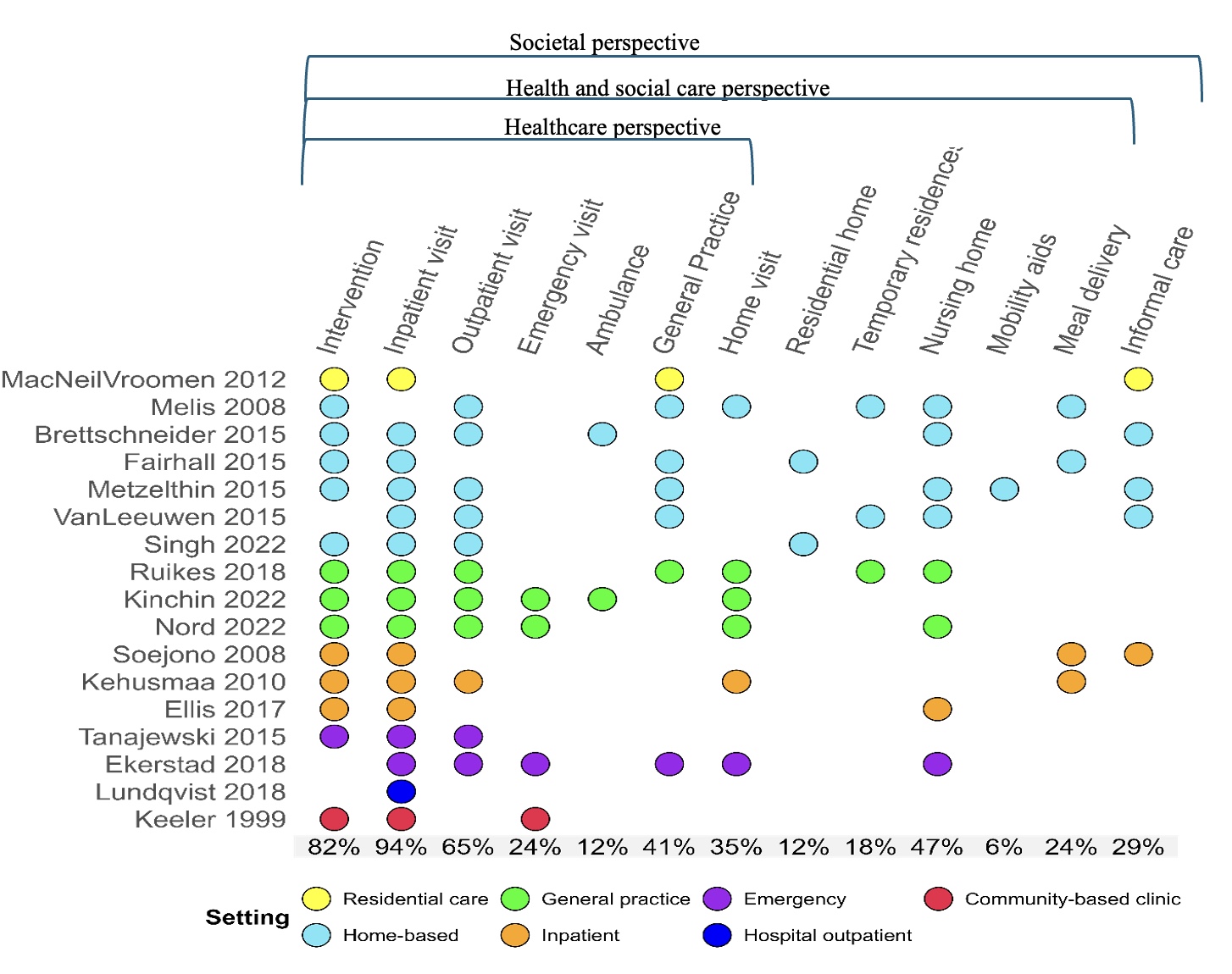


Figure S3: Measurements of health service utilisation for comparison groups by setting

Alt text: A plot showing healthcare, social care, and informal care resources included in cost estimation across Comprehensive Geriatric Assessment studies, grouped by economic perspective and care setting.


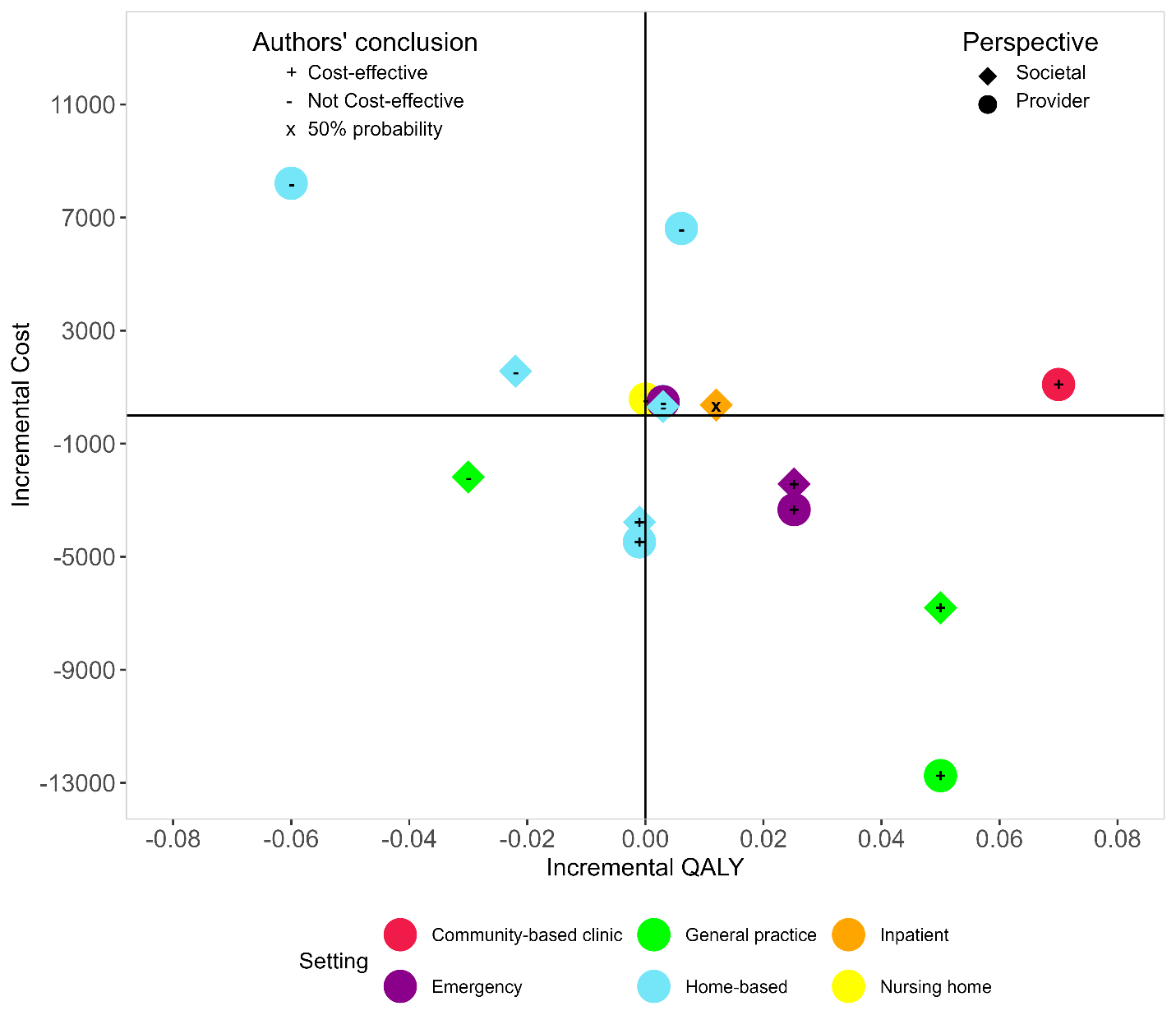


*One extreme value removed, please refer to* Table 3 *for full description of the studies.*

Figure S4: Cost-effectiveness plane for CGA intervention across settings compared to usual care, € 2024.

Alt text: Cost-effectiveness plane showing incremental costs and quality-adjusted life years of Comprehensive Geriatric Assessment compared with usual care across care settings.

Table S1: Characteristics of the studies

| Study ID | Country of study | Target population | Setting | Analytic approach | Perspective | Time horizon | Outcome | Instrument used to measure the outcome | Valuation of outcome |
| --- | --- | --- | --- | --- | --- | --- | --- | --- | --- |
| Kehusmaa 2010 | Finland | Age 65+ years, progressively decreasing functional ability, and risk of institutionalisation within 2 years | Inpatient | Trial based | Societal perspective | 12 months | Functional Independence Measure (FIM) | FIM^TM^ :A clinically significant improvement in FIM equals 22 points |  |
| VanLeeuwen 2015 | Netherlands | Frail population older than 65 | Home-based | Trial based | Societal perspective | 24 months | QALY | EQ 5D 3L | Dutch tariff |
|  |  |  |  |  |  |  | Health-related quality of life and functional limitation | Multiple measurement instruments |  |
| Kinchin 2022 | Australia | Community-dwelling older persons aged 70 or 50 and older with chronic conditions and complex needs. | General practice | Trial based | Health and societal care perpsective | 9 months | Functional Independence Measure | FIM: Point improvement |  |
|  |  |  |  |  |  |  | QALY | EQ-5D-3L and AQoL-8D | Australian preference weights |
| Fairhall 2015 | Australia | Frail community-dwelling people over 70 years with no cognitive decline. | Home-based | Trial based | Healthcare provider perspective | 12 months | Transition from frailty | Cardiovascular health study (CHS): Meeting fewer than 3 CHS frailty criteria at follow-up. |  |
|  |  |  |  |  |  |  | QALY | EQ-5D | UK tarrif |
| Metzelthin 2015 | Netherlands | Community-dwelling frail older people (older than 70 years) | Home-based | Trial based | Societal perspective | 24 months | QALY | EQ-5D | UK tariff |
|  |  |  |  |  |  |  | Disability | The Groningen Activity Restriction Scale |  |
| Keeler 1999 | USA | Older adults with at least one geriatric condition | Outpatient | Trial based | Not reported | 5 years | QALY | Quality of Well-Being (QWB) scale. | Not reported |
|  |  |  |  |  |  |  | Improvement in physical functioning | 10-item physical functioning scale from the RAND 36 item short form (SF-36) |  |
| Nord 2022 | Sweden | Individuals aged 75 years at high risk of hospitalisation | General practice | Trial based | Societal and healthcare provider | 24 months | QALY | EQ5D-3L | UK tariffs |
| Melis 2008 | Netherlands | Frail older patient older than 70 years | Home-based | Trial based | Healthcare provider perspective | 6 months | Successful treatment | *MOS-20MH and GARS-3: If MOS-20MH score increased by more than 10 points and the GARS-3 score declined no more than 4.5 points |  |
| Ellis 2017 | UK | Geriatric population above 65 | Inpatient | Model-based: decision analytic | Healthcare provider perspective |  | QALY | Barthel index | QALY: converting the Barthel index |
|  |  |  |  |  |  |  | Life year living at home (LYAH) | People living in their home |  |
|  |  |  |  |  |  |  | Life year gained |  |  |
| Singh 2022 | Scotland | Geriatric population considered for admission older than 65 | Home-based | Trial based | Health and social care perspective | 6 months | QALY | EQ-5D-5L | Crosswalk algorithm |
| Ruikes 2018 | Netherlands | Frail elderly aged above 70 years | General practice | Trial based | Healthcare provider perspective | 12 months | QALY | EQ-5D + C-3 L | Dutch tariff |
|  |  |  |  |  |  |  | Daily functioning | Katz-15 change score |  |
| MacNeilVroomen 2012 | Netherlands | Older adults with cognitive impairment | Long-term facilities | Trial based | Societal perspective | 6 months | QALY | SF-6D | SF6D tariff for QALY |
|  |  |  |  |  |  |  | Quality Indicator Score | Long-term Care Facility assessment: Sum score (lower score better quality of care) |  |
|  |  |  |  |  |  |  | Functional ability measure | COOP WONCA |  |
| Lundqvist 2018 | Sweden | People above 75 years with multimorbidity | Outpatient | Model-based: Markov model | Healthcare provider perspective | 2 years for the trial and 30 years for the model | QALY | EQ-5D-3L | UK tariff |
| Ekerstad 2018 | Sweden | Frail elderly patients, 75 years or older, in need of acute in-hospital treatment. | Emergency | Trial based | Societal perspective | 3 months | QALY | HUI-3 |  |
| Tanajewski 2015 | UK | Geriatric population aged 70 and over that are at risk of future health problems | Emergency | Trial based | Health and social care perspective | 3 months | QALY | EQ-5D-3L | Societal weights |
|  |  |  |  |  |  |  | Disability | Katz-ADL index: Point improvement |  |
| Brettschneider 2015 | Germany | Participants older than 80 years | Home-based | Trial based | Societal perspective | 18 months | QALY | EQ5D-3 L | EQ5D index scores from the UK |
| Soejono 2008 | Indonesia | Adults older than 60 years old and hospitalized due to geriatric syndrome | Inpatient | Model based: Decision tree | No statement | 30 days | Quality Adjusted Life Days | EQ5D |  |

*MOS-20MH: mental health subscale of the Medical Outcome; GARS-3: Groningen Activity Restriction Scale

Table S2: Valuation and measurement methods of informal care cost

| Valuation method | What is identified and measured | Advantages | Disadvantages |
| --- | --- | --- | --- |
| Opportunity cost method | Estimates the value of forgone benefit of caregiver due to informal care. | Reflects the real economic sacrifice made by the caregiver | Difficult to apply for retired, unemployed, or non-working carers |
| The proxy good method | Values the time spent on informal caregiving at a price close to market substitute. | Relatively simple to apply as the proxy value of each task can be estimate once and then used for different caregiving situations. | Assumes formal care and informal care are perfect substitutes. |
| Contingent valuation method | Measures how much caregivers would pay to avoid additional caregiving time, or the minimum amount of money caregiver would want to receive for providing additional caregiving time | Captures subjective burden and preferences; can reflect non-time aspects of caregiving burden | Hard to value time. In addition, stated intentions might be different from revealed behaviors. |
| Conjoint measurement | Measures caregiver preferences by asking respondents to trade off different attributes of caregiving, such as time, burden, flexibility, and payment | Can capture how caregivers value different aspects of caregiving simultaneously; useful for understanding preferences beyond time alone | Perceived to be cognitively demanding, especially older and less educated informal caregivers. |

Supplementary Text 1: Search strategy

**Medline (OVID)**

| 1. | Quality-Adjusted Life Years/ |  |  |  |  |
| --- | --- | --- | --- | --- | --- |
| 2. | Markov Chains/ |  |  |  |  |
| 3. | exp Models, Economic/ |  |  |  |  |
| 4. | cost*.ti. |  |  |  |  |
| 5. | (cost* adj2 utilit*).tw. |  |  |  |  |
| 6. | (cost* adj2 conse*).tw. |  |  |  |  |
| 7. | (cost* adj2 benef*).tw. |  |  |  |  |
| 8. | (cost* adj2 mini*).tw. |  |  |  |  |
| 9. | (cost* adj2 (effective* or assess* or evaluat* or analys* or model* or benefit* or threshold* or quality or expens* or saving* or reduc*)).tw. |  |  |  |  |
| 10. | (economic* adj2 (evaluat* or assess* or analys* or model* or outcome* or benefit* or threshold* or expens* or saving* or reduc*)).tw. |  |  |  |  |
| 11. | (qualit* adj2 adjust* adj2 life*).tw. |  |  |  |  |
| 12. | QALY*.tw. |  |  |  |  |
| 13. | (incremental* adj2 cost*).tw. |  |  |  |  |
| 14. | ICER.tw. |  |  |  |  |
| 15. | utilities.tw. |  |  |  |  |
| 16. | markov*.tw. |  |  |  |  |
| 17. | (dollar* or USD or cents or pound or pounds or GBP or sterling* or pence or euro or euros or yen or JPY).tw. |  |  |  |  |
| 18. | ((utility or effective*) adj2 analys*).tw. |  |  |  |  |
| 19. | (willing* adj2 pay*).tw. |  |  |  |  |
| 20. | ((euroqol or euro-qol or euroquol or euro-quol or eurocol or euro-col) adj3 ("5" or five)).tw. |  |  |  |  |
| 21. | (EQ-5D* or EQ5D*).tw. |  |  |  |  |
| 22. | (european* adj2 quality adj3 ("5" or five)).tw. |  |  |  |  |
| 23. | 1 or 2 or 3 or 4 or 5 or 6 or 7 or 8 or 9 or 10 or 11 or 12 or 13 or 14 or 15 or 16 or 17 or 18 or 19 or 20 or 21 or 22 |  |  |  |  |
| 24. | geriatric assessment/ |  |  |  |  |
| 25. | ((integrated adj3 (care or model)) and (elderly or old* or geriatric)).tw. |  |  |  |  |
| 26. | comprehensive geriatric assessment.mp. |  |  |  |  |
| 27. | ((geriatric or elderly or aged) adj3 (consultation* or evaluation* or assessment*)).tw. |  |  |  |  |
| 28. | 24 or 25 or 26 or 27 |  |  |  |  |
| 29. | 23 and 28 |  |  |  |  |

**EMBASE (ovid)**

| 1 | 'geriatric assessment'/exp OR 'geriatric assessment' |
| --- | --- |
| 2 | 'geriatric assessment'/exp OR 'comprehensive geriatric assessment':ti,ab,kw OR ((integrated NEAR/3 (care OR model)) AND (elderly OR old OR geriatric)) |
| 3 | (((geriatric?:ti,ab OR elder*:ti,ab OR old:ti,ab) AND age:ti,ab OR old*:ti,ab) AND adult?:ti,ab OR senior?:ti,ab OR old*:ti,ab) AND ((patient? NEXT/5 (assess* OR evaluat* OR consult*)):ti,ab) |
| 4 | 'geriatrics'/exp |
| 5 | 'evaluation and follow up'/exp |
| 6 | #4 AND #5 |
| 7 | #1 OR #2 OR #3 OR #6 |
| 8 | 'economic evaluation'/exp OR 'cost-utility analysis'/exp OR 'cost-utility analysis' OR 'cost-effectiveness analysis'/exp OR 'cost utility analysis'/exp OR 'resource allocation'/exp |
| 9 | (cost* NEAR/2 (effective* OR utilit* OR benefit* OR minimi* OR unit* OR estimat* OR variable* OR value OR efficienc* OR minimi?ation)):ab |
| 10 | 'economic evaluation*':ti,ab OR icer:ti,ab OR cua:ti,ab OR 'resource allocation':ti,ab OR budget*:ti,ab |
| 11 | (economic* NEAR/1 (evaluation* OR analysis OR model*)):ti,ab |
| 12 | economic*:ti OR cost*:ti |
| 13 | #8 OR #9 OR #10 OR #11 OR #12 |
| 14 | #7 AND #13 |
| 15 | #7 AND #13 |

**CINAHL**

| S1 MM "Geriatric Assessment" |
| --- |
| S2 (MH "Geriatric Assessment+") |
| S3 (MH "Health Services for the Aged") |
| S4 TI (integrated N3 (care OR model)) AND (elder* OR old* OR geriatric?)) |
| S5 TI ("comprehensive geriatric assessment*") OR AB ("comprehensive geriatric assessment*") |
| S6 TI ((geriatric? or elder* or old age or old* adult? or senior? or old* patient?) N5 (assess* or evaluat* or consult*)) OR AB ((geriatric? or elder* or old age or old* adult? or senior? or old* patient?) N5 (assess* or evaluat* or consult*)) |
| S7 (MH "Health Status+") |
| S8 (MH "Aged+") |
| S9 7 and 8 |
| S10 S1 OR S2 OR S3 OR S4 OR S5 OR S6 OR S9 |
| S11 TI ( cost* N2 (effective* OR utilit* OR benefit* OR minimi* OR analy* OR conse*) ) OR TI ( value N2 (money) ) OR TI ( markov OR monte carlo ) OR TI ( decision* N2 (tree* OR analy* OR model*)) OR AB ( cost* N2 (effective* OR utilit* OR benefit* OR minimi* OR conse*) OR AB ( value N2 (money) ) OR AB ( markov OR monte carlo ) OR AB ( decision* N2 (tree* OR analy* OR model*) ) |
| 12 S10 AND 11 |

**NHSEED**

comprehensive geriatric assessment [any field]

geriatric assessment [title]

geriatric evaluation [title]

geriatric consultation[title]

**Tuft’s CEA registry (basic search)**

Geriatric assessment OR geriatric

Comprehensive geriatric assessment

(article.title:"integrated" OR article.title:"care") AND article.title:"old"
